# Identification of immunosuppressive neutrophils using multi-omics: why functional testing remains key

**DOI:** 10.3389/fimmu.2026.1814355

**Published:** 2026-05-18

**Authors:** Fleur van Oosterom, Angela A. F. Gankema, Felipe Rojas-Rodriguez, Arie J. Hoogendijk, Taco W. Kuijpers

**Affiliations:** 1 Department of Molecular Hematology, Sanquin Research, Amsterdam University Medical Center, University of Amsterdam, Amsterdam, Netherlands; 2 Department of Pediatric Immunology, Rheumatology and Infectious Disease, Emma Children's Hospital, Amsterdam University Medical Center, University of Amsterdam, Amsterdam, Netherlands

**Keywords:** cancer, neutrophil, PMN-MDSCs, proteomics, RNAseq, MDSC activity

## Abstract

Polymorphonuclear neutrophils (PMNs) are innate cells that may act as myeloid-derived suppressor cells (MDSCs), most often characterized by their immunosuppressive capacity towards immune cells within the tumor microenvironment. Over the last decade, many studies have tried to characterize these PMN-MDSCs. Although all human neutrophils obtain suppressive activity upon activation, specialized subsets, including low-density cells, have been proposed to exert MDSC activity without requiring prior stimulation. Single-cell RNA sequencing (scRNAseq) has created a new horizon to study possible neutrophil subsets. As such, the identification of distinct neutrophil subtypes in tumors may better characterize immunosuppressive neutrophils, in the end leading to specific targeting strategies in cancer patients. Nevertheless, scRNAseq has not yet led to a clear immunophenotypic characterization of PMN-MDSCs, and clinical relevance by functional testing is still lacking. Studies on the discrepancy in RNA abundance, protein expression and functionality in neutrophils indicate that the future for defining functional neutrophil subsets will rely on further development of single-cell techniques, like proteomics, to truly carve out neutrophil subsets and plasticity.

## Introduction

Polymorphonuclear neutrophils (PMNs) are the most abundant circulating leukocyte in human blood (1). Armed with various effector functions necessary for pathogen clearance, they play a crucial role in innate immune defense (1). Neutrophils have long been overlooked in research due to the challenges that they bring, for example, their short lifetime and easy activation upon isolation (2, 3). With novel omics approaches to study cells in more detail, the interest in studying neutrophils has also increased. Developments in the cancer field have highlighted a paradoxical role for neutrophils as crucial contributors to an immunosuppressive environment that is established in many cancers (1, 4).

Tumor growth is supported by a highly specialized environment consisting of fibroblasts, blood vessels, local extracellular protein meshwork, and immune cells, the so-called tumor microenvironment (TME) (5). Although the presence of neutrophils in the TME has been known for decades, this was not taken too much notice of. However, it is now recognized that inflammation and neutrophils play a critical role at multiple stages of cancer: initiation, growth, metastasis, and response to therapy (6).

Furthermore, cancer is associated with elevated circulating neutrophil counts, high neutrophil-to-lymphocyte ratios, and increased neutrophil infiltration in multiple solid tumors, all of which correlate with a worse prognosis (7, 8). This is further emphasized by Gentles et al. (9) who by assessing gene expression signatures of immune cells in approximately 18,000 human tumors, observed that intratumoral ‘neutrophil mass’ was the most significant adverse cancer-wide prognostic marker in 39 different malignancies (9).

To understand the relationship between neutrophil load and prognosis, the dual role of neutrophils in cancer has been studied. On the one hand, neutrophils may have anti-tumor characteristics, for example, by directly killing tumor cells or via antibody dependent cellular cytotoxicity (ADCC) (10, 11). While on the other hand, neutrophils contribute to tumor growth by promoting angiogenesis, metastasis, as well as by creating an immunosuppressive environment amongst others by the recruitment of regulatory T cells (Tregs) and by inhibition of T cell proliferation and activation. The latter is often referred to as myeloid-derived suppressor cell (MDSC) activity (1, 12–15). This activity was previously suggested to be exerted by a subset of neutrophils, dubbed PMN-MDSCs (16). To better understand, and eventually eradicate, PMN-MDSC activity in cancer, the field is currently trying to define the origin and identify specific markers of these cells.

Since isolation of enough intact and pure PMN-MDSCs from human tumors is highly challenging for many technical reasons, neutrophil phenotypes in cancer have been explored by methods for which cell isolation is not required, like RNA sequencing, mass spectrometry, and metabolomics. The use of these approaches has led to multiple putative PMN-MDSC specific markers (17, 18). Moreover, RNA sequencing indicated a considerable neutrophil transcriptional plasticity (18). A classification system for the identification of neutrophils by single-cell RNA sequencing (scRNAseq) has finally been proposed which is suggested to help decrease ambiguity in the many descriptions of subpopulations, including PMN-MDSCs (19).

In this review, we will focus on neutrophil function in relation to phenotype, density, and subsets. This will be followed by highlighting the development of scRNA-seq and single cell proteomics for identifying neutrophil subsets, particularly the identification of PMN-MDSCs with a focus on the suggested cell surface markers. Next, the value of RNA signatures and the incorporation of additional proteomics data will be discussed in the light of functional plasticity and current avenues advancing the field of neutrophil-centered therapeutics.

## Immunosuppressive low-density neutrophils

Isolation of circulating neutrophils is commonly performed using density gradient separation. The average density of neutrophils from healthy individuals is higher than lymphocytes and monocytes (20), thereby enabling effective separation by ending up as normal density neutrophils (NDNs) in the red cell pellet. However, identification of a population of neutrophils in the lymphocyte fraction led to the subpopulation dubbed as low density neutrophils (LDNs) (21–26). These LDNs were shown to have immunosuppressive capacity whereas NDNs do not (26–28). Later studies found accumulation of LDNs with cancer progression (29–31). However, the opposite has also been described (13). Both LDNs and NDNs have recently been shown to have immunosuppressive capacity (17, 32).

Importantly, neutrophil density is to a large extent due to the granular composition and/or cellular age. For instance, blood kept overnight will result in more neutrophils ending up in the ring fraction, and even subtle pre-activation *in vivo* or unforeseen activation following the initial steps of cell purification could already reduce density (33, 34). A recent study showed that LDNs could be obtained *in vitro* by stimulating NDNs with N-formyl-methionyl-leucyl-phenylalanine (fMLP) (26). When studied in healthy granulocyte donors pre-stimulated with G-CSF the day before, the number of neutrophils steeply increased, and part of the immature cells had the typical LDN features, which helped to compare LDNs and NDNs in more detail (15). This again showed that both LDNs and NDNs from healthy donors possess immunosuppressive characteristics (35). Therefore, density is not a key determinant factor in the identification or subtyping of PMN-MDSCs.

## Context-dependent neutrophil plasticity

Based on this phenotypical and functional distinction, the obvious question is what gives rise to this suppressive behavior: nature or nurture? In other words, is this behavior locally induced or programmed during neutrophil differentiation?

**Figure 1:**
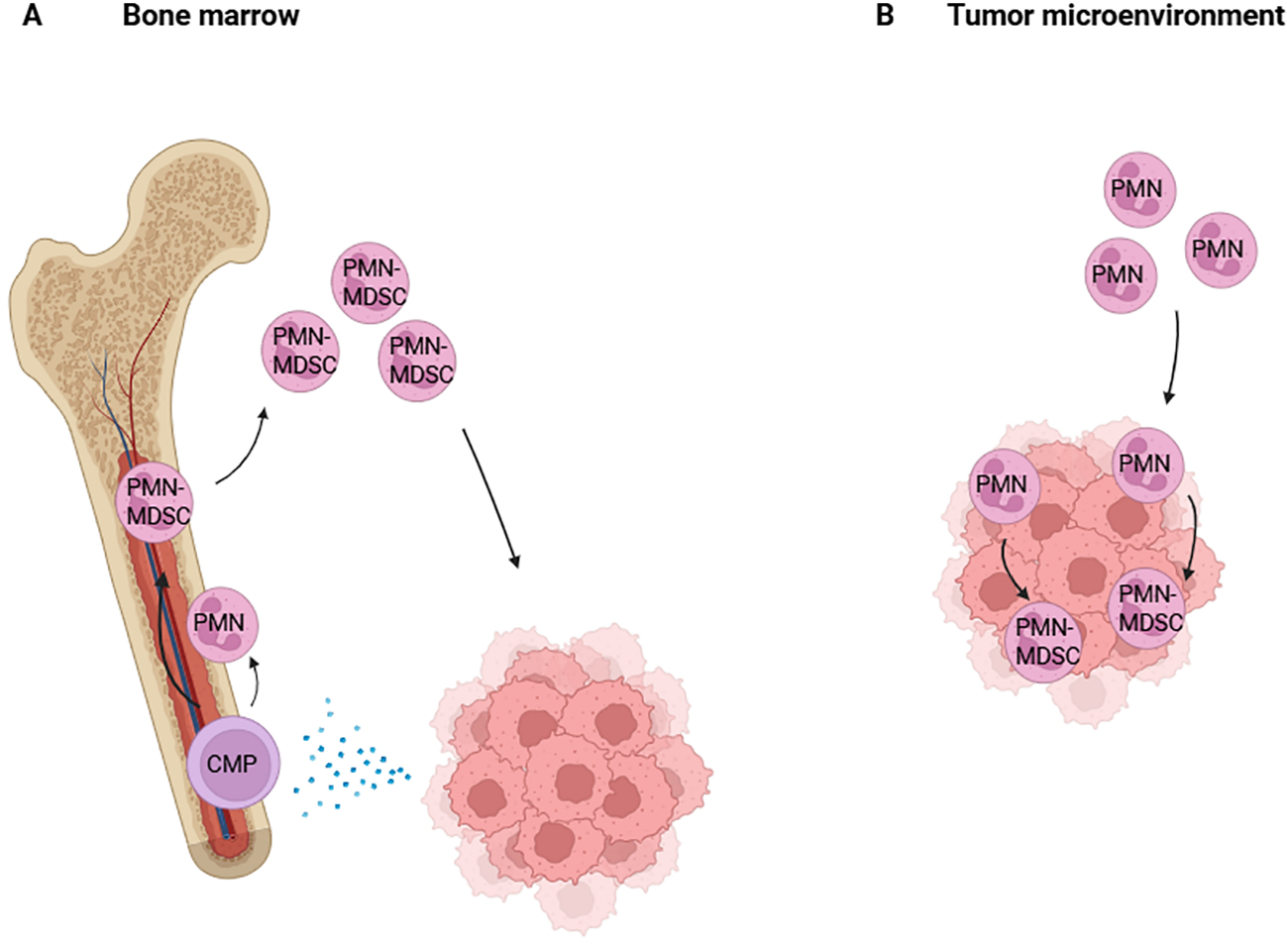
Two non-mutually exclusive hypotheses on the induction of PMN-MDSC activity. In **(A)** tumor cells produce different factors acting at a distance on myeloid progenitors in the bone marrow. Some studies suggest that these tumor-derived factors alter myeloid differentiation and reprogram immature myeloid cells into PMN-MDSCs with immunosuppressive functions. In **(B)** local factors in the TME induce the immunosuppressive capacities of neutrophils that have been attracted to infiltrate the tumor mass. This shows neutrophil plasticity to adapt and become PMN-MDSCs, which will strongly depend on the local TME.

**Figure 2:**
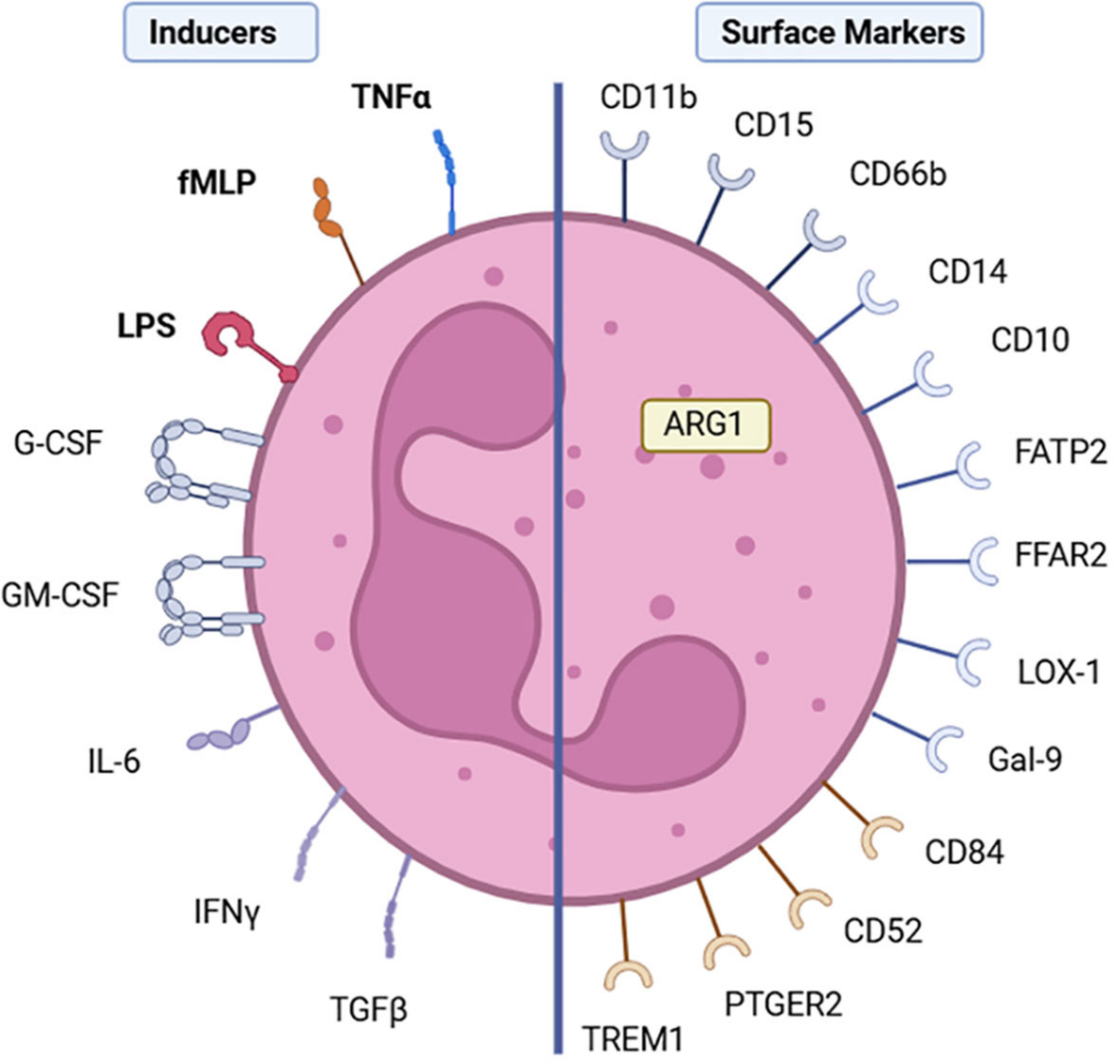
Suggested inducers of human PMN-MDSC activity leading to upregulation of cell surface markers, or corresponding transcripts, and ARG1. The different stimuli that have been described in literature to induce MDSC activity in neutrophils and the markers that have been described in literature to be able to distinguish PMN-MDSCs from inflammatory neutrophils. Colored receptors for neutrophil-MDSC inducers are confirmed in humans, while the grey ones are from mouse data. Surface proteins depicted in grey are based on data acquired on protein level. Surface proteins suggested by single cell RNA sequencing signatures are depicted in orange.

A recent study on mammary carcinoma suggested skewing of hematopoiesis towards myelopoiesis from the time that the tumor started to develop, inducing immunosuppressive programming of neutrophils during their differentiation from progenitor cells in the bone marrow (36). Combined single-cell, chromatin and functional analyses in these mouse models showed tumor-derived factors like interleukin-1 beta (IL-1β) drive the reprogramming of neutrophils in the bone marrow at the hematopoietic stem and progenitor cell (HSPC) level (37, 38). Here, reprogramming during differentiation could potentially enable immature neutrophils to become PMN-MDSCs instead of mature PMNs (17) (**Figure 1**). However, we and others have shown that early reprogramming is not essential for PMNs to obtain MDSC activity. Various soluble factors can activate all neutrophils isolated from blood of healthy donors to exert MDSC activity. In our hands, a strong and reproducible inducer is TNFα, and to a lesser extent autolytic and microbial substances fMLP and LPS (13, 39). Other studies have suggested similar effects with G-CSF, GM-CSF, IL-6, TGFβ and IFNy stimulation of murine neutrophils (16, 40, 41) (**Figure 2**), which have not been confirmed with human neutrophils, possibly marking a relevant species difference (42).

Plasticity in biology is described as the ability to acquire distinct phenotypes and function in response to local signals (43). As such, the change of neutrophils from NDN to LDN by fMLP stimulation as well as the induction of PMN-MDSC activity with TNFα, fMLP or LPS shows that neutrophils have a certain level of plasticity. This plasticity could be appreciated more like a continuum of different cell states instead of subsets, constantly fine-tuning protein levels to its immediate surroundings by protein release or shedding of surface proteins (44).

It has also been postulated that neutrophils exhibit an inflammatory, anti-tumor function at the edge of the tumor, and upon migrating to the tumor core become immunosuppressive (45). In the TME, a neutrophil is subjected to cytokines excreted by the tumor, for example the immunosuppressive cytokine TGFβ, resulting in reprogramming of neutrophils towards immunosuppressive neutrophils (46) (**Figure 1**). These PMN-MDSCs are known to inhibit T cell proliferation and activation, as well as stimulate tumor growth (1, 12, 14, 15). Blocking TGFβ, by using the receptor inhibitor SM16, reversed this phenotype and showed an increase in inflammatory neutrophils (46).

Another example of neutrophil plasticity has been shown in acute tissue injuries. The presence of proinflammatory molecules, such as TNFα and LPS, leads to hyper-activated neutrophils showing increased reactive oxygen species (ROS) production and release of their nuclear DNA as Neutrophil Extracellular Traps (NETs), during a cell death program called NETosis (47, 48). However, during the resolution phase of inflammation, neutrophils with anti-inflammatory characteristics arise, which inhibit T cell proliferation (49, 50). Thus, neutrophil plasticity may play a crucial role in both the initiation and in the resolving phase of inflammatory disease.

One cell marker identifying specific neutrophil subsets has been acknowledged, glycoprotein CD177 (51, 52). CD177, was described to modulate neutrophil migration by the regulation of chemotactic receptor expression and by mediating integrin activation (53). CD177 is localized in the specific granules. The proportion of CD177^+^ neutrophils per individual differs but remains stable over time. However, in pregnancy, sepsis, and some pathological conditions, the population of CD177^+^ neutrophils increases (3, 54). This increase in CD177^+^ neutrophils is reversed when the pathological condition is solved, showing plasticity even in defined subsets. The function of CD177 on neutrophils is incompletely understood and distinct immunological roles of CD177^-^ and CD177^+^ neutrophils have not yet been established (3). Nevertheless, in tumors, an increase in CD177^+^ neutrophils is positively associated with overall survival and disease-free survival (55–57). While the above-mentioned feature does show differences in neutrophils in any given individual, the question of whether these subsets are biologically relevant remains unanswered.

## Uncertain specificity of PMN-MDSC markers

Regarding MDSC nomenclature, PMN-MDSCs are repeatedly defined as CD11b^+^CD14^−^CD15^+^ or CD11b^+^CD14^−^CD66b^+^ cells (**Figure 2**) (58). In general, several proteins have been suggested to associate with PMN-MDSC activity in human and mice (**Table 1**). However, these surface markers are mostly associated with the maturation or activation status of neutrophils and are therefore expressed by neutrophils in general (13, 59, 72). Since neutrophils seem to change their phenotypes in response to activation within the blood stream or to their surroundings, distinct surface markers related to the immunosuppressive character of neutrophils in the TME have been reported, including CD10 and CD14 (59, 60), which may be merely upregulated by cellular activation. Therefore, a clear distinction between PMN-MDSCs and pro-inflammatory neutrophils is not possible by only using these markers.

**Table 1:** Candidate PMN-MDSC markers and their validation.

Candidate marker	Reference	Model system used	Disease or cell line	Defined by	Validation in clinical setting
CD10	59	Human	G-CSF treated donors, SLE	Flow cytometry, T cell proliferation, T cell cytokine secretion	In patients with lymphoma, solid tumors and SLE similar CD10^-^ and CD10^+^ neutrophil populations were identified
CD14	60	Mice	LLC, EL4 lymphoma, KPC	scRNAseq, cell mass spectrometry, flow cytometry, suppression assay	–
		Human	NSCLC	bulk RNAseq, scRNAseq, CyTOF	Similar to mouse PMN-MDSCs, human tumor PMNs showed upregulation of CD14
FATP2	61	Mice	LLC, EL4 lymphoma, CT26 colon carcinoma	LC-MS lipidomics analysis, whole genome RNA sequencing, qPCR, Western Blot, suppression assay	*Slc27a2-/-* mice showed a decrease in tumor growth and lost the ability to suppress antigen-specific CD8^+^ T cell responses. The pharmacologic inhibition of FATP2 abrogated the activity of PMN-MDSCs and delayed tumor progression
		Human	HNC, NSCLC, BC	LC-MS lipidomics analysis	–
FFAR2	62	Mice	LLC, B16F10	Real time qPCR, RNAseq, T cell proliferation assay	*ffar2-/-* mice showed a reduction in MDSCs and decreased tumor growth
		Human	Adenocarcinoma	Immunofluorescent staining	–
LOX-1	63	Human	NSCLC, HNC, CC, MM	Whole gene expression array, flow cytometry, suppression assay, immunofluorescent microscopy	The number of LOX-1^+^CD15^+^ PMN-MDSCs in CC, HNC, and NSCLC increased 8-10 fold. OLR-1 expression and the presence of LOX-1^+^ PMN-MDSCs are associated with clinical parameters
PD-L1	64	Human	HCC	Flow cytometry	Decreased levels of PD-L1^+^ MDSCs in treated HCC patients correlated with significantly longer disease free survival
	65	Human	Sepsis	Flow cytometry, T cell proliferation assay	–
Gal-9	66	Mice	EL4, 4T1	T cell proliferation, T cell cytokine secretion, tumor growth	Galectin-9 transgenic mice showed a marked decrease in the ability to prime Th1 immune responses and generate effector or memory T cells. TIM-3 expression on T cells increased the number of PMN-MDSCs
	67	Human	NPC	RNAseq, immunofluorescent staining, ELISA	High concentrations of tumor and plasma Gal-9 are associated with shortened survival of NPC patients
CD84	17	Human	NSCLC, HNC and G-CSF treated donors	scRNAseq, flow cytometry	–
CD52					
PTGER2					
TREM1	68	Human	19 different cancer types	scRNAseq, spatial transcriptomics, T cell activation by IFNy and TNFα secretion	High TREM1 expression was found to be associated with a poor prognosis in various cancer types
ARG1*	12	Human	Healthy donors	Arginase activity assay, T cell proliferation, ion exchange chromatography	A patient with complete arginase I deficiency showed no arginine deprivation and no inhibition of T cell proliferation
	69	Mice	3LL	Immunofluorescent staining, T cell proliferation, tumor growth	Injections of specific arginase inhibitors, including non-hydroxy arginine, resulted in a dose-dependent inhibition of tumor growth
		Human	RCC	Arginase activity assay, western blot, plasma levels of arginine measurement, T cell proliferation	–
	70	Human	NSCLC	Immunohistochemistry, arginase activity assay, T cell proliferation	Patients with NSCLC have increased ARG1 plasma levels compared to healthy controls
	59	Human	G-CSF treated donors	Real time qPCR, arginase activity assay, T cell proliferation	–
	71	Human	Pemphigus	RNAseq, T cell proliferation	–
	1	Human	Endometrial cancer	scRNAseq, immunofluorescent staining,	Higher percentages of VISTA^+^ARG^+^ MDSCs were associated with worse disease free survival and overall survival

*is an enzyme that has been specifically indicated for PMN-MDSC activity.

BC, Breast cancer; CC, Colon Cancer; HCC, Hepatocellular carcinoma; HNC, Head and Neck Cancer; LC-MS lipidomics, Liquid chromatography-mass spectrometry lipidomics; LLC, Lewis lung carcinoma; MM, Multiple Myeloma; NSCLC, Non Small Cell Lung Cancer; RCC, Renal cell carcinoma; NPC, Nasopharyngeal carcinoma; SLE, Systemic Lupus Erythematosus.

The hunt for specific PMN-MDSC markers continued by including differences in maturation markers since immunosuppressive neutrophils have mostly been described as an immature subset of neutrophils (15, 73). However, a study performed with G-CSF-treated donors showed that CD10^high^ (mature) and CD10^low^ (immature) neutrophils differ in their immunosuppressive ability in circulation with most immunosuppressive activity in the mature CD66b^+^CD10^high^ neutrophils displaying an activated phenotype (59). These mature neutrophils inhibited *in vitro* T cell proliferation and interferon γ (IFN-γ) production via CD18-mediated contact-dependent arginase 1 (ARG1) release, which is considered a key feature of their suppressive nature. Importantly, the study argued against the concept that a more immature nature would endow these cells with a more outspoken suppressive character.

Two other surface markers that have been alluded to as being present on PMN-MDSCs only are Fatty Acid Transport Protein-2 (FATP2) and Free Fatty Acid Receptor 2 (FFAR2), both related to fatty acid recognition (61, 62). The main mechanism of FATP2-mediated suppressive activity involves the uptake of arachidonic acid and the synthesis of prostaglandin E2 (PGE2) (61). Mouse and human PMN-MDSCs were reported to exclusively upregulate FATP2. This overexpression of FATP2 in PMN-MDSCs was shown to be controlled by GM-CSF, activating the transcription factor STAT5 (61). In a mouse model, deletion of FATP2 decreased the suppressive activity of PMN-MDSCs. Furthermore, selective pharmacologic inhibition of FATP2 also abrogated the activity of PMN-MDSCs and substantially delayed tumor progression when combined with checkpoint inhibitors in another experimental mouse model (61). A role for FATP2 in human immunosuppression by neutrophils still needs to be confirmed.

FFAR2, also named GPR43, is a surface expressed G protein-coupled receptor for short-chain fatty acids (SCFAs) with a high degree of homology between mouse and human (74). The role of SCFAs in neutrophil functioning includes migration, cytokine production, and generation of ROS (75). Metabolite profiling revealed accumulation of acetic acids in tumor tissues in both cancer patients and a cancer mouse model. In this study, FFAR2 was found to be highly expressed in the myeloid cells of lung adenocarcinoma patients, which may suggest the impact that SCFAs have by inducing MDSC activity (62). In mice, myeloid *ffar2* gene deletion markedly inhibited urethane-induced lung carcinogenesis and syngeneic tumor growth. An overall reduction in MDSCs and increased CD8^+^ T cell infiltration was observed, which was linked to reduced expression of ARG1. This inhibition could be relieved by L-arginine replenishment or inhibition of PPAR-γ (62). In humans, the influence of these SCFAs on PMN-MDSC activity has not yet been investigated.

Furthermore, expression of lipoprotein receptor-1 (LOX-1), a lectin that acts as receptor for oxidized low-density lipoprotein, has been shown to discriminate PMN-MDSCs from neutrophils without this suppressive capacity (63). However, we and others have shown that expression of LOX-1 on neutrophils does not mean these neutrophils are immunosuppressive (42). These cells have a pro-inflammatory role in allergic rhinitis instead (76). Expression of LOX-1 on neutrophils cultured from CD34^+^ HSPCs, while not showing immunosuppressive activity, was not any different from that on activated blood neutrophils that did suppress T cell function (39). For that reason, LOX-1 expression cannot distinguish PMN-MDSCs considering it can be instantly upregulated from the intracellular granules upon cell activation (13).

The expression of PD-L1 on neutrophils has also been implied in the inhibition of T cell responses (64, 65). T cells express PD-1, which is upregulated upon exhaustion (77). When PD-1 interacts with PD-L1, T cell function and proliferation are inhibited (78). Multiple studies looking into neutrophils from patients with sepsis showed that neutrophils with immunosuppressive features show a higher level of PD-L1 expression (65, 79). Subsequently, blocking the PD-1/PD-L1 axis with avelumab, an anti-PD-L1 neutralizing antibody, increased *in vitro* T cell proliferation of sepsis patients (65). Another study on human MDSC subsets in cancer, infection, and inflammation showed that upregulation of PD-L1 was mostly observed for monocytic MDSCs (M-MDSCs) and not on PMN-MDSCs (80). Whether the use of avelumab has targeted M-MDSCs instead of PMN-MDSCs remains an intriguing possibility.

Galectin-9 (Gal-9), member of the β-galactoside-binding animal lectins family, is described to be involved in PMN-MDSC activity too (81). In its extracellular form, it can favor the interaction with T cells via binding to TIM-3, leading to its immunosuppressive activity (81). Gal-9 was first described in transgenic murine models leading to increased numbers of both M-MDSCs and PMN-MDSCs (66). In a tumor model, both intra-cellular and extra-cellular Gal-9 were shown to promote neutrophil immunosuppressive activity in the TME (67). Moreover, high amounts of Gal-9 were found in the tumor tissue and serum from tumor-bearing patients, which are negatively associated with disease outcome (67).

More recently, scRNAseq has been performed on tumor-derived neutrophils and G-CSF-treated donor neutrophils to identify PMN-MDSC specific markers. CD52, CD84, and the prostaglandin E2 receptor (PTGER2) transcripts were identified to mark PMN-MDSCs (17). In this study, mature NDNs isolated from tumor tissue were dubbed mPMN-MDSCs. On these isolated cells, transcript levels of CD52, CD84, and PTGER2 were generally increased compared to circulating neutrophils. However, immunosuppressive activity of mPMN-MDSCs expressing these surface proteins was not validated in a T cell proliferation assay to check T cell inhibition. In addition, scRNAseq data from neutrophils in 19 different cancer types revealed an immunosuppressive cluster of neutrophils high in Triggering Receptor Expressed on Myeloid cells 1 (TREM1). These TREM1^high^ neutrophils were enriched in chemotaxis and oxidative stress pathways, while TREM1^low^ neutrophils were enriched in cytoplasmic translation and ribosome biogenesis pathways (68). Additionally, an increase in TREM1^high^ neutrophils was associated with more immunosuppression. However, TREM1^high^ cells are described as a subpopulation of PMN-MDSCs, which implies that not all PMN-MDSCs would have increased TREM1 expression. Furthermore, the sample size for certain cancers was limited. Therefore, further studies still need to validate generalizability of the cell population identified. These data suggest that the scRNAseq findings may not be PMN-MDSC specific, continuing the hunt for PMN-MDSC markers.

## Proteomic neutrophil states in different settings

Advances in mass spectrometry using optimized sample preparation methods, innovations in separation techniques, and ultra-low-flow nano liquid-chromatography have improved sensitivity to study protein abundance at single cell level (82). If isolation of sufficient cells for flow cytometry is not feasible or inaccurate, single cell proteomics by mass spectrometry (SCoPE-MS) is a method using a carrier protein and isobaric labeling for the identification of low abundant proteins for validation of scRNAseq data (82–84). To date, single cell proteomics on neutrophils remains challenging because of the relatively low protein content in these cells (85). However, a recent mass spectrometry-based study compared circulating mature CD10^high^ LDNs, circulating immature CD10^low^ LDNs to NDNs and tumor associated neutrophils (TANs) derived from glioblastoma in mini-bulk proteomics (500 cells) and single cell proteomics (86). For single cell proteomic analysis >1,100 proteins were identified per single neutrophil. Based on the most variable protein features amongst the different groups that were compared, seven distinct neutrophil populations were identified which differed in surface markers (CD11b/CD18 and CD66b) and granule proteins (myeloperoxidase, elastase and cathepsin G). Differences in granule content cannot have been picked up by transcriptomic analysis, since production of these proteins happens at early stages during myeloid development in the bone marrow (87). The corresponding transcripts might already be long gone, while the protein is still present, being stored within the azurophilic or specific granules. Notwithstanding, the granule content is highly relevant since neutrophil effector functions can be directly related to the release of toxic proteins from these granules upon neutrophil activation (88). With neutrophils, which are known to have a very low amount of RNA transcription going on as end-stage short-lived cells, protein data may be more important for inferring clinical or functional relevance.

Sample preparation for single cell methods may lead to contamination by plasma proteins or platelets. For example, CD52, as part of the immunosuppressive PMN-MDSC signature (17) has been shown to be expressed in platelets too, while proteomic validation is lacking (89, 90). Moreover, our own results in human primary neutrophils cultured under serum-free conditions from HSPCs show no CD52, while CD84 (also expressed by platelets) is indeed expressed on these cells (39). Endocytosis of RNA or protein by platelets or neutrophils, while not generating the protein themselves at any stage of their formation, might be another reason to be cautious not to overinterpret scRNAseq data without further verification steps. Finally, another limitation in single cell analysis is in the analysis itself that often starts with a predefined population of neutrophils. For example in the abovementioned study on CD52, CD84 and PTGER2, the scRNAseq signature was defined in PMN-MDSCs based on the markers CD66b^+^CD10^low^CD11b^high^CD16^high^, but for their PMN-MDSC signature not all markers were required to be present at the same time, allowing cells co-expressing only one or two of these markers as possible contaminants (17). Therefore, it would be essential that single cell techniques are independently validated using both *in vitro* and *in vivo* models.

## Discrepancies in neutrophil RNA and protein content

While in the cancer field, single cell multi-omics has hugely propelled the knowledge on tumor heterogeneity and cell states (91–93), in the immunology field the interpretation of the technique is less straight-forward because of the plasticity and mismatch between transcript and protein levels. In many studies, genome-wide correlation levels between mRNA and proteins in immune cells are around 40% (94, 95). These discrepancies have mostly been ascribed to the mode of action of immune cells (96, 97). They are required to be fast responders and therefore, often have a high RNA content which is unstable but can be stabilized quickly when necessary, or they store proteins in granules (98).

Particularly in case of neutrophils, RNA abundance does not correlate with protein expression and cellular function (39, 87). Moreover, a study on proteomics and transcriptomics on circulating neutrophils showed that the proteome is dominated by four highly expressed proteins, neutrophil defensin 3A, alarmin S100A8, lysozyme, and cathepsin G, while the transcriptome is dominated by *MYCBP*, *KIF2C*, and *ATP6V0B* (99). No correlation was found between the proteome and transcriptome data. This can be due to changes in rates of RNA degradation or protein synthesis, as well as long term storage of proteins in the granules when RNA is already degraded (100). The transcriptome might also reflect the readiness for some specific transcripts of naive neutrophils to initialize gene expression upon activation, but do not show any protein content yet (86).

A recent single-cell multi-omics study using nanoSPLITS (nanodroplet splitting for linked-multimodal investigations of tracesamples) has further highlighted the discrepancy at the transcript and protein level in primary cells (101). This was the first study that performed both single cell RNAseq and single cell proteomics on the same cell. The distribution of correlations between transcript-protein pairs from a single cell was only modestly different to a distribution of randomly sampled correlations. While the aim of that study was not to clarify discrepancies between RNA and protein content, it is further evidence that the two do not correspond.

Single cell proteomics enables the identification of possible subsets based on protein expression directly. Therefore, developing a method that integrates single cell RNAseq and proteomics would be ideal. Cellular Indexing of Transcriptomes and Epitopes by Sequencing (CITE-seq) is an example of the integration of RNAseq and protein expression (102). However, this technique is still mostly limited to surface proteins by using a labeled antibody for the detection of proteins. Intracellular CITE-seq is being developed (103), but a major limitation remains the need for a specific antibody. Unbiased proteomics using this technique is not yet possible. Moreover, for intracellular CITE-seq, permeabilization and cell fixation are required, which mostly negatively impact RNA assays (104).

All these ‘omic’ techniques show a snapshot of a cell in a certain state or environment at the time of analysis. Integration of all these snapshots together over different time frames gives the best indication of the cell phenotype. However, it is not possible yet to track single cell phenotypical changes over time, hampering the examination of neutrophil plasticity in time.

## The development of single cell metabolomics

Single cell metabolomics is yet another single cell omics technique that has undergone quick development in the past few years. Metabolomic analysis uncovers the chemical phenotype of a cell (105). While single cell metabolomics has not yet been performed on neutrophils, metabolic differences between neutrophils and PMN-MDSCs in bulk have been identified. Similar to cancer cells, PMN-MDSCs rely on glycolysis and produce lactate (106, 107). In a murine model in which glucose availability was restricted, a decrease in MDSC activity was observed when splenic neutrophils were co-cultured with T cells (107). Furthermore, the hypoxic TME has been suggested to affect PMN-MDSC phenotype and metabolism (108). Under hypoxic conditions, HIF1α is stabilized, while being degraded under normal conditions (109). HIF1α translocates to the nucleus where it dimerizes with HIF1β and acts as a transcription factor for a number of genes, including genes involved in glucose transport and metabolism (109, 110). While mostly relying on glycolysis, fatty acid oxidation can also be used as an energy source (111). Inhibition of fatty acid oxidation decreased the immunosuppressive capacity of MDSCs in a mouse model bearing 3LL tumors (112). However, no distinction between M-MDSCs and PMN-MDSCs was made. Optical metabolic imaging has been performed on neutrophils as well before and showed rapid metabolic changes in neutrophils during activation, leading to a reduced redox state (113).

Single cell metabolomics would reveal more detailed information on a cell’s state. Currently, single cell metabolomics is mostly used in assessing drug uptake and drug metabolism to look at possible drug resistance (114, 115). Another new development is single-cell spatial metabolomics with cell-type specific protein profiling (116, 117). This technique allows the incorporation of untargeted spatial metabolomics and targeted multiplexed protein imaging in a single pipeline. This has been done for both tumor cells as well as B and T cells. However, this has not been performed on neutrophils yet.

## Identification of PMN-MDSCs by functional activity

Functional assays remain the gold standard for defining neutrophil immunosuppressive capacity in clinical studies. These assays complement current scRNAseq and proteomic analyses by helping delineate which fractions to include when characterizing the molecular features of PMN-MDSCs and clarifying how they differ from inflammatory neutrophils.

Two findings are noticeable in routine *in vitro* assays to measure PMN-MDSC activity. First, neutrophil activation is required to induce this so-called *in vitro* T cell suppressive activity, and this may be relevant to interpret the data on cellular density, phenotype, and pre-activation of PMN-MDSCs. Secondly, neutrophils from both controls and treatment-naive cancer patients behaved similarly, had normal density and were without spontaneous MDSC activity (13). Whether this type of *in vitro* PMN-MDSC activity is identical to the immunosuppression in the TME itself is unclear (118).

**Figure 3:**
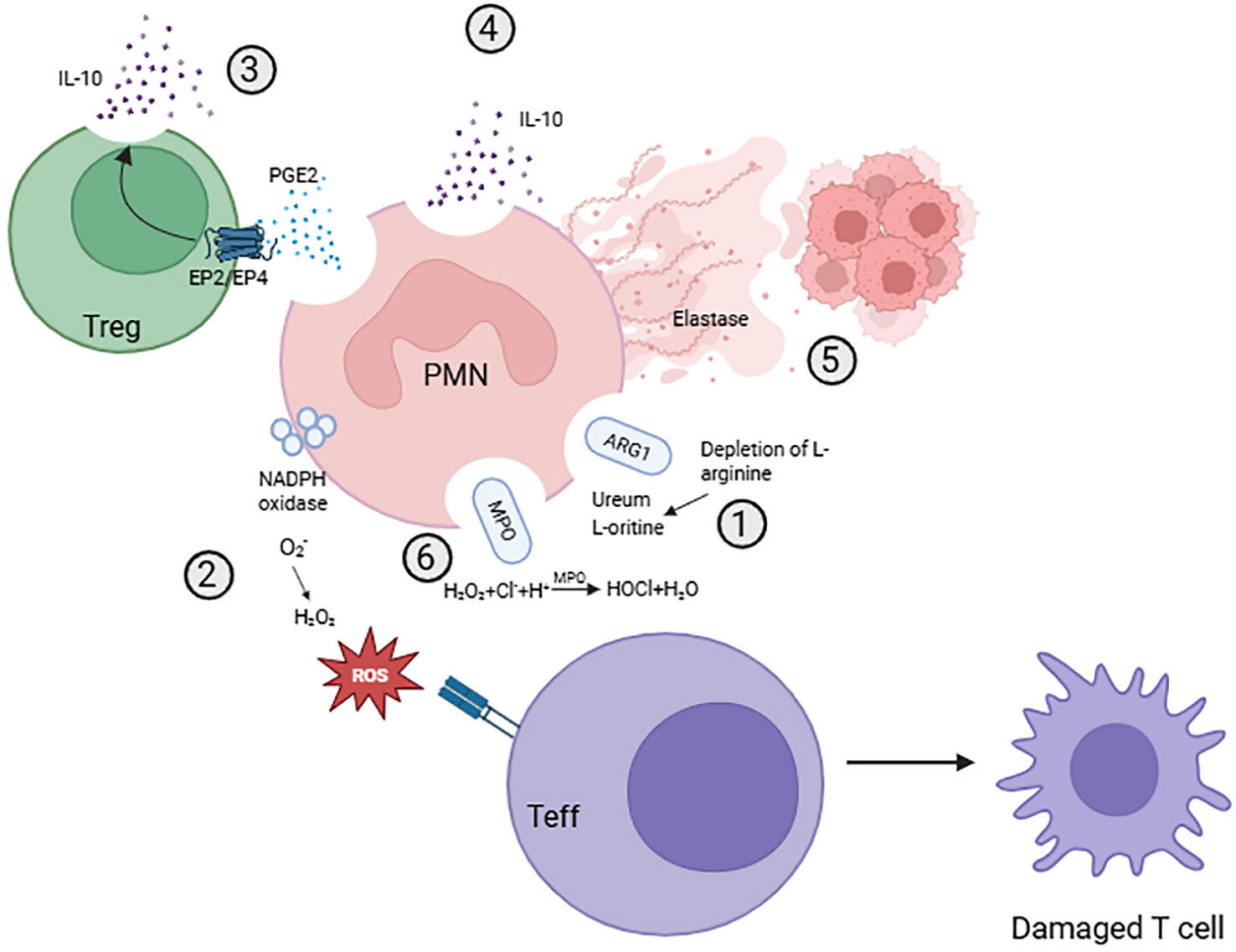
Suggested mechanisms of human PMN-MDSC activity. Different mechanisms suggested for the inhibition of T cells by PMN-MDSCs. (1) Release of ARG1 into the extracellular space between T cells and neutrophils depletes L-arginine from the environment, which is necessary for T cell proliferation. (2) The production of reactive oxygen species (ROS) by neutrophils, which leads to downregulation of the T cell receptor. (3) Secretion of PGE2 by neutrophils which can bind EP2/EP4 receptors on Tregs, promoting Treg expansion as well as suppressive function via induction of IL-10 production (4) Secretion of anti-inflammatory cytokines, like IL-10, stimulating the suppressive activity of Tregs and dampening the proliferation and activation of effector T cells. (5) NETosis leading to the release of proteases and shielding of tumor cells from cytotoxic T cells. (6) Degranulation leading to the release of the peroxidase MPO, which can convert hydrogen peroxide produced by the NADPH oxidase complex to the oxidant hypochlorous acid.

One enzyme has been suggested to drive PMN-MDSC activity: i.e. the aforementioned ARG1 (12, 69–71). During development of human neutrophils in the bone marrow, ARG1 becomes expressed and stored in specific granules (87, 119). L-arginine is an important source for T cell proliferation, which gets eliminated by extracellular ARG1 release upon neutrophil activation (**Figure 3**) (119). This makes ARG1 a potentially interesting candidate to explain immunosuppression, although inhibition of ARG1 activity by the chemical inhibitor nor-NOHA rescues T cell proliferation and suppresses tumor growth in some but not all experimental models (12, 69, 120, 121).

The lack of consistency in ARG1 data raises the question whether other mechanisms in humans contribute to the immunosuppressive activity of neutrophils. The secretion of anti-inflammatory cytokines, like IL-10 (71, 122), has also been described to be involved in T cell suppression by inhibiting the production of pro-inflammatory cytokines and dampen the activation and proliferation of effector T cells (123). PMN-MDSCs have also been suggested to recruit regulatory T cells, via chemotactic factors like PGE2, to the TME (124). This promotes Treg expansion and also stimulates IL-10 production, which consequently stimulate the suppressive activity of Tregs and dampens T effector cell proliferation and activation (123). Research also supports *de novo* development of regulatory T cells by factors like IL-10 (124).

Furthermore, NETosis has been suggested to play a role in carcinogenesis, both directly and indirectly (125). NETs in lung and pancreatic cancers have been shown to release matrix metallopeptidase 9, which can directly promote cancer growth by degradation of the extracellular matrix, stimulating angiogenesis and metastasis (126). A more indirect role of NETs has been described for the inhibition of T cell activation. NETosis may lead to shielding and coating of tumor cells protecting them from T cell cytotoxicity (127). Furthermore, with the release of NETs, other neutrophil factors and proteases are also released into the extracellular space, for example myeloperoxidase and elastase, which could also be detrimental to T cell functioning by itself, as such NETosis has been linked to T cell exhaustion in the TME (128).

As supported by our recent data on HSPC-derived neutrophils, the data thus far show that PMN-MDSCs may not phenotypically differ from circulating PMNs although there is a definite distinction between immunosuppressive and inflammatory neutrophils in function (39).

## PMN-MDSCs and CAR-T cell therapy

Chimeric antigen receptor (CAR)-T cell therapy has proven very successfully as a cellular immunotherapy for the treatment of B cell malignancies that do not respond to conventional treatment strategies (129). In contrast, when neoantigens could be used for targeting, CAR-T cells for the treatment of solid tumors were less successful and only led to partial responses, mostly likely due to the immunosuppressive TME (130, 131). PMN-MDSCs may contribute to the limited efficacy of CAR-T cell therapy in solid tumors. For instance, neuroblastoma patients receiving GD2 CAR-T cell therapy and who relapsed showed enrichment of circulating PMN-MDSCs (132). CAR-T cells cultured with PMN-MDSCs derived from neuroblastoma patients led to a decrease in CAR-T cell survival and proliferation, while neutrophils from healthy donor peripheral blood did not (132). However, the definition of PMN-MDSCs and how they were isolated is unclear from this paper. In a murine study with highly cytotoxic GD2 CAR-T cells against GD2 expressing tumor cells *in vitro*, GD2 CAR-T cells had no effect against GD2^+^ sarcoma, while still controlling neuroblastoma (133). It was shown that the GD2^+^ sarcoma model resulted in the induction of a large population of both M-MDSCs and PMN-MDSCs, which was not found for neuroblastoma. Combining GD2 CAR-T cell therapy with all trans retinoic acid (ATRA) improved the efficacy by depleting M-MDSCs and diminishing the suppressive activity of PMN-MDSCs, indicating that MDSCs were responsible for the decreased CAR-T cell efficacy 133).

On a different note, to enhance CAR-T cell therapy, targeting antigens shared by tumor cells and MDSCs are being explored, for example TRAIL-R2 (also known as DR5 or TNFRSF10B) (134, 135). Engagement of this receptor with its antigen resulted in caspase 8-mediated apoptosis (134). Co-expressing TRAIL-R2 and HER2 on CAR-T cells significantly enhanced CAR-T cell therapy efficacy against orthotopic tumors established in mouse models (136). Although considered promising, TRAIL-R2 agonistic antibodies have failed at the (pre)clinical stage, which may affect the efficacy of TR2 CAR-T cell therapy as well (137, 138). Moreover, protective immunity may be affected as well since the expression of TRAIL-R2 is also upregulated by activated B lymphocytes (139). A more specific PMN-MDSC target would be favorable to enhance CAR-T cell therapy. These results again show the importance of further characterizing PMN-MDSCs.

## PMN-MDSC activity beyond cancer: a role in preventing autoimmunity?

Immunosuppressive neutrophils are not only observed in cancer settings, PMN-MDSCs have also been reported to accumulate in secondary lymphoid organs of patients with a variety of autoimmune diseases such as inflammatory bowel disease, systemic lupus erythematosus, multiple sclerosis, rheumatoid arthritis, and autoimmune hepatitis (140–144). Moreover, PMN-MDSCs have been described to increase during infectious diseases like sepsis or severe COVID-19 caused by SARS-CoV-2, but often solely based on phenotypic or transcriptomic description (141, 145) and only rarely tested for their immunosuppressive capacity *ex vivo* (146). Irrespective of whether all normal neutrophils in the blood or once reprogrammed at the stage of HSPCs are able to exert so-called PMN-MDSC activities, these findings suggest that the immunosuppressive behavior of neutrophils represent a natural reaction to protect from and avoid injury in the host by steering away from prolonged and potentially auto-destructive processes.

## Summary

While research into the immunosuppressive function of neutrophils has increased, specific identification of PMN-MDSCs is still lacking since isolation of neutrophils from tumor tissue without further activation is still a major issue. To circumvent this issue, researchers have been focusing on using scRNAseq signatures to investigate neutrophil plasticity and possibly identify neutrophil subtypes, if present at all. However, these scRNAseq signatures need to be used with caution, since discrepancies between transcriptomics, proteomics and function have been shown, especially for neutrophils.

While many developments in the ‘omic’ field are taking place, there are still challenges to overcome. One of the major challenges is the low abundance of surface proteins, making it difficult to detect them in bulk analysis and even undetectable with mini-bulk or single cell proteomics. This limitation also highlights the need for further optimalization and an increased coverage of immune signaling proteins. Additionally, identifying post-translational protein modifications remains a challenge due to low stoichiometry, while post-translational protein modifications may be highly relevant for cell functioning.

Furthermore, accumulation of a certain metabolic molecule does not always correlate with the metabolic activity in the cell. If a molecule accumulates, it may correlate with decreased consumption. A method for tracking metabolite abundance of a single cell over time would be required to investigate the metabolic activity of a single cell, which is rather unrealistic in case of tissue sampling.

Overall, we conclude that the lack of specific neutrophil subsets, fixed states or markers of immunosuppressive conditions continues to exist. The question arises whether a single marker or signature would be ever able in humans to distinguish in absolute terms the immunosuppressive from inflammatory capacity of neutrophils. Instead, PMN-MDSCs in cancer tissue may be judged more as an induced functional activity rather than the representation of a fixed state or specific subset. Therefore, the focus on identifying such cells by function would be more relevant but comes with great technical challenges to isolate neutrophils from tumor tissue to test this activity. Development of single-cell proteomics and metabolomics are promising methodologies that may help determining this activity. Although it is impossible to estimate its role compared to current cancer treatment options (including chemotherapy, radiation, cellular immunotherapy and/or checkpoint inhibitors) any future interference with the immunosuppressive activity of neutrophils will provide more insight into the role and contribution of so-called PMN-MDSC activity in cancer or inflammatory disorders. If the mechanisms behind PMN-MDSC activity are better understood and can be shown to be highly relevant in human disease conditions like cancer, targeting these PMN-MDSC activity specifically could be beneficial for cancer patients while promoting this immunosuppressive activity of neutrophils in autoimmune diseases. This could ultimately lead to better intervention strategies for both cancer patients as well as patients with autoimmune disease.

## Glossary

ADCC: Antibody Dependent Cellular Cytotoxicity
ARG1: arginase1
CAR-T cell: chimeric antigen receptor T cell
CITE-seq: Cellular indexing of Transcriptomes and Epitopes by Sequencing
FATP2: Fatty Acid Transport Protein-2
FFAR2: Free Fatty Acid Receptor 2
fMLP: N-formyl-methionyl-leucyl-phenylalanine
G-CSF: granulocyte colony-stimulating factor
GM-CSF: granulocyte and monocyte colony-stimulating factor
HSPC: hematopoietic stem and progenitor cell
IFN-γ: interferon γ
IL-1β: interleukin-1 bèta
IL-10: interleukin-10
LDN: Low density neutrophil
LOX-1: lectin-like oxidized low-density lipoprotein receptor-1
MDSC: myeloid-derived suppressor cell
NDN: normal density neutrophil
NETs: Neutrophil Extracellular Traps
PGE2: prostaglandin E2
PMN: polymorphonuclear neutrophil
PTGER2: prostaglandin E2 receptor
ROS: reactive oxygen species
SCFA: short-chain fatty acids
SCoPE-MS: single cell proteomics by mass spectrometry
scRNAseq: single cell RNA sequencing
TAN: tumor associated neutrophil
TME: tumor microenvironment
TREM1: Triggering Receptor Expressed on Myeloid cells 1.
Candidate marker: Model system(Disease/cell line)CD10 (57), Human (G-CSF treated donors, SLE)
CD14 (58): Mice (LLC, EL4 lymphoma, KPC)
Human (NSCLC): RNAseq, scRNAseq, CyTOF
FATP2 (59): Mice (LLC, EL4 lymphoma, CT26 colon carcinoma)
Human (HNC: NSCLC, BC), LC-MS lipidomics analysis
FFAR2 (60): Mice (LLC, B16F10)
Human (Adenocarcinoma): Immunofluorescent staining
LOX-1 (61): Human (NSCLC, HNC, CC, MM)
PD-L1: Human (HCC
63): Flow cytometry, T cell proliferation assay
Gal-9: Mice (EL4, 4T1
65): RNAseq, immunofluorescent staining, ELISA
CD84: CD52 and PTGER2 (16), Human (NSCLC, HNC and G-CSF treated donors)
TREM1 (66): Human (19 different cancer types)
ARG1*: Human (Healthy donors
69): RNAseq, T cell proliferation
67): Arginase activity assay, western blot, plasma levels of arginine measurement, T cell proliferation
68): Immunohistochemistry, arginase activity assay, T cell proliferation
57): Real time qPCR, arginase activity assay, T cell proliferation
1): scRNAseq, immunofluorescent staining,
67): Immunofluorescent staining, T cell proliferation, tumor growth.
